# Using Non-supervised Artificial Neural Network for Determination of Anthropogenic Disturbance in a River System

**DOI:** 10.21315/tlsr2017.28.2.14

**Published:** 2017-07-31

**Authors:** Nurul Ruhayu Mohd Rosli, Khairun Yahya

**Affiliations:** 1 Centre for Marine and Coastal Studies, Universiti Sains Malaysia, 11800 USM Pulau Pinang, Malaysia; 2 School of Biological Sciences, Universiti Sains Malaysia, 11800 USM Pulau Pinang, Malaysia

**Keywords:** Anthropogenic Activities, Non-Supervised Artificial Neural Networks, Physical and Chemical Parameters

## Abstract

The study of river water quality plays an important role in assessing the pollution status and health of the water bodies. Human-induced activities such as domestic activities, aquaculture, agriculture and industries have detrimentally affected the river water quality. Pinang River is one of the important rivers in Balik Pulau District that supplies freshwater for human consumption. A total of 442 physical and chemical parameters data of the Pinang River, Balik Pulau catchment were analysed to determine the sources of pollutants entering the river. Non-supervised artificial neural network (ANN) was employed to classify and cluster the river into upstream, middle-stream and downstream zones. The monitored data and non-supervised ANN analysis demonstrated that the source of nitrate was derived from the upper part of the Pinang River, Balik Pulau while the sources of nitrite, ammonia and ortho-phosphate are predominant at the middle-stream of the river system. Meanwhile, the sources of high total suspended solid and biological oxygen demand were concentrated at the downstream of the river.

## Abstract (ru)

Kajian kualiti air sungai memainkan peranan yang penting dalam menilai status pencemaran dan kesihatan air. Aktiviti manusia seperti domestik, akuakultur, pertanian dan industri telah menjejaskan kualiti air sungai. Sungai Pinang adalah salah satu sungai penting di daerah Balik Pulau, yang membekalkan air tawar untuk kegunaan manusia. Parameter fizikal dan kimia di kawasan tadahan Sungai Pinang, Balik Pulau dianalisis untuk menentukan sumber bahan pencemaran yang memasuki sungai. ‘artificial neural network’ (ANN) telah digunakan untuk mengkelas dan mengkluster sungai kepada bahagian hulu, tengah dan hilir. Data yang dipantau dan analisis ANN menunjukkan bahawa sumber nitrat berasal dari bahagian hulu Sungai Pinang, Balik Pulau, manakala sumber nitrit, ammonia dan orto-fosfat mempunyai pengaruh besar di bahagian tengah sistem sungai. Sementara itu, jumlah tinggi pepejal terampai dan keperluan oksigen biologi tertumpu di hilir sungai.

## INTRODUCTION

Pinang River, one of the important rivers in Balik Pulau district in Malaysia, is heavily impacted by anthropogenic activities such as domestic activities, aquaculture and agriculture (DOE, 2008). The creeks flowing into the Pinang River, Balik Pulau receive untreated wastewater discharge directly from small villages situated along the river. The anthropogenic activities existing at the river upstream eventually affects the water quality of the river and may have serious impacts, in terms of water pollution, on the marine ecosystem.

The Pinang River, Balik Pulau catchment area supplies freshwater to more than 53,000 people in the Penang Island of Malaysia. Thus, it is particularly important to ensure that the water is safe for drinking. The guidelines for drinking water as proposed by DOE (2008) and WHO (2008) are important efforts to monitor any source of pollution that endangers human health. Therefore, the objective of this study was to identify the sources of pollutants that enter into the Pinang River, Balik Pulau.

## MATERIALS AND METHODS

### Sampling Sites

**Figure 1:**
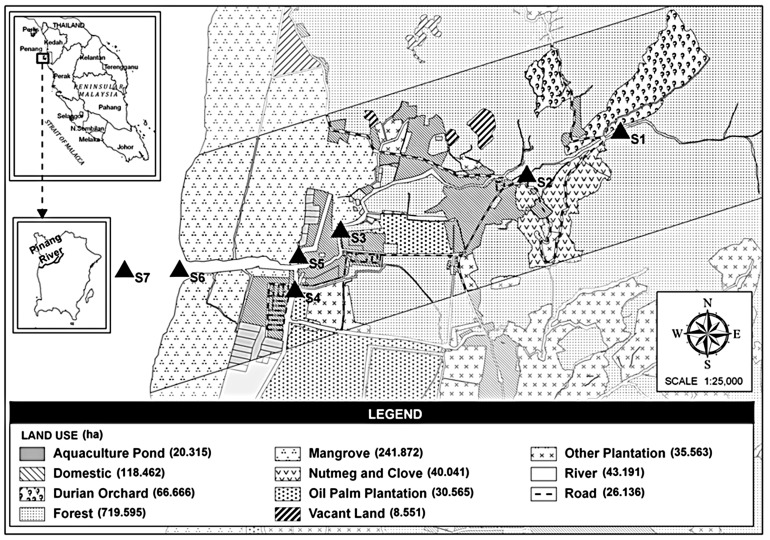
Variation of land-use activities along the Pinang River, Balik Pulau at the sampling stations with the Global Positioning System (GPS) coordinates.

Pinang River is located in Balik Pulau district in the north-western part of Penang Island between coordinates 5°23′26.71″N 100°10′40.62″E and 5°24′12.79″N 100°13′36.01″E. It is a shallow river of 6.5 km in length with a depth of 0.13–3.03 m and a width of 2–80 m. The Penang Water Authority pump house is located at the upper part of the river and is responsible for collecting and supplying the freshwater to the residents of Balik Pulau. Water samples were collected from seven stations along the flow of the river, commencing from the upstream until the downstream end, into the sea (Fig. 1 and Table 1). Sampling was done twice a month from October 2007 to October 2008 during spring and neap tides at low and high tides. During the spring tide, the river experiences a strong tidal mixing in the water column with a greater length of seawater intrusion compared to that observed during neap tide. This may show variation in water quality parameters for both tidal events.

### Physical and Chemical Parameters

The parameters like temperature (°C), pH, dissolved oxygen (DO) (mg/L), salinity (ppt) and electrical conductivity (EC) (μMHOS) were measured *in-situ* at the sampling stations. The collected data for DO and temperature were measured using DO Meter YSI Model 57. Dissolved salts and ions for water EC level was determined by S-C-T Meter (YSI). Hydrogen ion concentration (pH) and salinity were assessed using portable battery-operated pH (EUTECH Instruments) and hand refractometer (ATAGO), respectively.

However, the determination and analysis of parameters like total suspended solid (TSS), mg/L, nitrite (μM), nitrate (μM), ammonia (μM), ortho-phosphate (μM) and biochemical oxygen demand (BOD_5_), mg/L were made in the laboratory. TSS was obtained by filtration of water samples by using Whatman Glass Fibre Filter GF/C with the particle retention of 0.45 μm. Nutrient determination for nitrite concentration was measured by diazotisation with sulphanilamide and its reaction with N-(1-napthyl)-ethylenediamine to form a highly azo dye colour (Adams 1990). For nitrate analysis, the water samples were required to pass through the reduction process to develop nitrite form. The process required a column that contained cadmium filings coated with metallic copper (Adams 1990). This procedure involved in attaining the actual nitrate concentration in water samples with subtracted the calculated nitrite concentration given. Phenol-hypochlorite method was used to measure ammonia concentration (Strickland & Parsons, 1972). The sample is mixed with alkaline citrate and sodium hypochlorite solutions and phenol in the presence of sodium nitroprusside, which act as a catalyser, to form a blue indophenol with ammonia concentration (Strickland & Parsons, 1972). For orthophosphate determination, the blue solution of ortho-phosphate concentration is developed from the complex heteropoly acid due to the reaction between a reagent compound containing ammonium molybdate, sulphuric acid, ascorbic acid and trivalent antimony (Boyd & Tucker 1992). BOD_5_ of 300 mL sampled water was measured from the concentration of oxygen consumed by microbial activities following totally dark incubation at 20°C for 5 days (APHA 1985). A total of 442 data on physical and chemical parameters were collected.

### Non-supervised ANN

**Figure 2:**
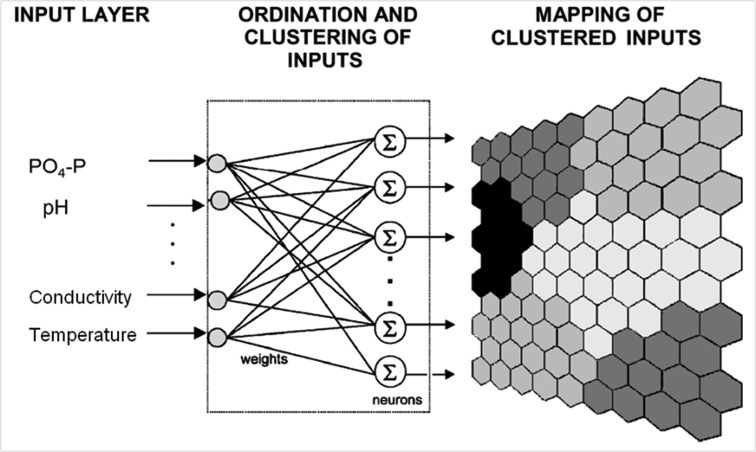
The Self-Organizing Map (SOM) algorithm.

The Self-Organizing Map (SOM) algorithm has been derived from the non-supervised artificial neural network (ANN) concept, which was developed by Kohonen (1984). This algorithm was applied to the ordination, clustering, and mapping of physical and chemical parameters data by categorising the Pinang River, Balik Pulau into upstream, middle-stream and downstream zones. The principal approach is presented in a simplified manner as shown in Figure 2. The non-supervised ANN consists of neurons that classify between similar and dissimilar features of the normalised input data. Thus, it can be mapped into clustered input forms (Recknagel *et al*., 2006). Those features are calculated between the similarity of the inputs and weights of Euclidean distances that can be visualised and partitioned by the unified distance matrix (U-matrix) and partitioned map (K-means) (Vesanto *et al*. 2000; Kalteh *et al*. 2008).

**Figure 3:**
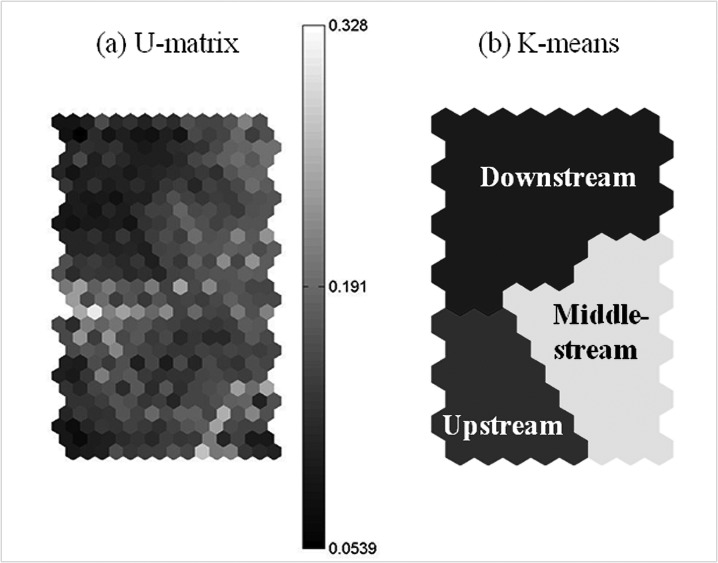
The zoning of clusters for Pinang River, Balik Pulau from October 2007 until October 2008.

Figure 3 shows the zoning of clusters for Pinang River, Balik Pulau from October 2007 until October 2008 as mapped according to Table 2 by the U-matrix and K-means partitioning using the SOM Toolbox of Matlab 6.5 (2002). The U-matrix map (Fig. 3(a)) represents the relative distances between neighbouring data of the input data space as shades of grey. The lighter areas in the U-matrix visualise the smallest distances between neighbouring data to indicate regions or clusters. The dark colour represents neighbouring data with the largest distances and denotes the borders between clusters. The K-means algorithm partitions the input data space into a specified number of clusters based on the U-matrix (Recknagel *et al*. 2006; Chan *et al*. 2007). Figure 3(b) visualises the corresponding partitioned map for the three zones at Pinang River, Balik Pulau.

There are 11 input variables including temperature, pH, DO, BOD_5_, salinity, EC, TSS, nitrite, nitrate, ammonia and ortho-phosphate that were used for ordination and clustering by means of the SOM Toolbox for Matlab 6.5.

## RESULTS AND DISCUSSION

**Figure 4:**
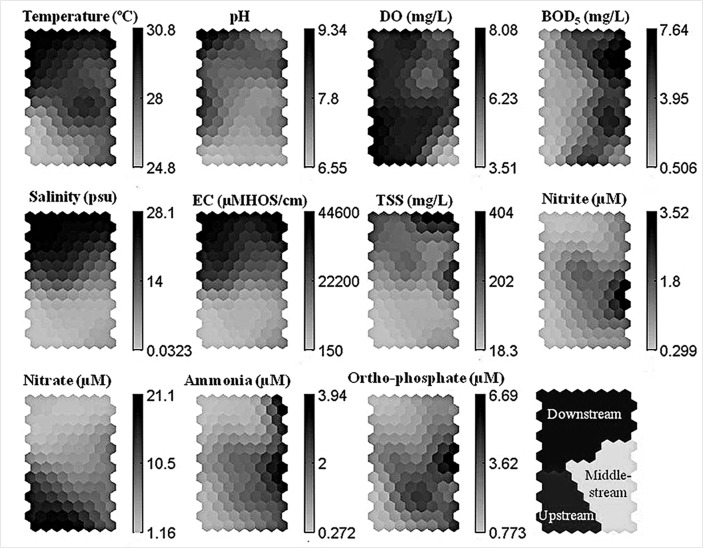
The ordination and clustered data using a non-supervised ANN tool.

Table 2 presents the water quality parameters (minimum, maximum and mean value) measured at all the stations along the Pinang River, Balik Pulau. The ordination and clustered data using a non-supervised ANN tool is shown in Figure 4.

The low mean water temperature (24.4°C) at upstream was due to the hilly landscape that was covered with forests and orchards. The unsheltered environment of the flowing river demonstrated a slightly higher water temperature at middle-stream (28.5°C) and downstream ends (29.6°C). Higher water temperatures near the sea could heat up the shallow water from the coast of mangrove estuary tidal flats (Sanderson & Taylor 2003). Thus, watershed vegetation cover plays an important role in determining the temperature of the stream water whereas seasonality in rainfall and stream discharge are less defined (Ramírez *et al*., 2014).

The pH values of the river were in the range of 4.7–9.8. Based on WHO (2008), the optimum pH value for drinking water is in the range of 6.5–9.5; however, the pH of 6.5–8.5 was annotated by DOE (2008). A slightly higher pH values above 9.5 was recorded irregularly along the middle-stream and downstream; this may be due to the photosynthesis algae activities that consume CO_2_ dissolved in water (Drische *et al*., 2008). Occasionally, lower pH was recorded at the upstream (Station 2) and middle-stream (Station 3). This may be due to the acidic discharge and fertiliser usage from the agriculture run-off (Sanjay Kumar *et al*. 2006) and domestic sewage (Mendiguchía *et al*. 2007) into the river.

The DO level varied from 6.3–9.1 mg/L at the upstream, 1.0–10.5 mg/L at the middle-stream, and 1.7–10.6 mg/L at the downstream of the river. A few measurements (about 6 times) indicated the very low level of DO at Station 6. This was probably due to the high content of organic pollutants from oil palm plantation leachates (Sanjay Kumar *et al*. 2006) and aquaculture discharges (Mirzoyan *et al*. 2008) that allowed the bacterial utilisation of DO during the respiration process.

All the sampling stations measured high BOD_5_ with an exception at Station 1. The minimum and maximum values of BOD_5_ in the upstream, middle-stream and downstream ranged from 0–6.8 mg/L, 0–12.6 mg/L, and 0–13.9 mg/L, respectively. A high BOD_5_ level was recorded on certain sampling days, especially at middle-stream (4.1 mg/L) followed by the downstream (3.7 mg/L). Flowing discharges of untreated domestic sewage from the upstream to the downstream were the main causes of BOD_5_ deterioration in the river ecosystem (Johnson *et al*. 2002; Uzoukwu *et al*. 2004; Sanjay Kumar *et al*. 2006).

A certain part of the Pinang River, Balik Pulau experienced tidal fluctuation, which reached until the middle-stream (Station 3) with salinity 31 ppt. EC recorded 10–35 μMHOS at upstream, 20–48000 μMHOS at middle-stream and 210–50000 μMHOS at downstream. In general, elevated EC concentrations in the water bodies were influenced by high salinity levels, especially during the spring tide. During this tidal event, the bottom part of the water frequently exhibited higher levels of salinity and EC compared to the surface water as reported by Gao *et al*., (2008). The highest salinity value during spring and neap tides were 33 ppt and 32 ppt, respectively. Not much variation in tidal amplitude occurred during neap tide. The convergence of TSS downstream indicated a higher mean of TSS (244.3 mg/L). However, the upstream (14.9 mg/L) and middle-stream (136.9 mg/L) predominantly indicated low and moderate TSS. High TSS at Station 5 was due to the effluent discharge from aquaculture ponds that consisted of uneaten pellets used for prawn, fish and livestock. In addition, high precipitation is another factor that contributed to the increase in TSS (Mendiguchía *et al*. 2007), which transported suspended solids from the upstream towards the downstream.

The concentrations of nitrite at upstream, middle-stream and downstream were in the range of 0–1.2 μM (mean 0.3 μM), 0–18.1 μM (mean 1.6 μM) and 0–19.6 μM (mean 0.9 μM), respectively. At certain sampling days, high nitrite concentration was observed in surface water (Stations 3, 5 and 6), especially during the low tide of spring tide. The elevated nitrite could be from domestic discharges and aquaculture effluents. Agricultural land at stations 1, 2 and 4 did not contribute to the high level of nitrite. In this study, the nitrite levels did not exceed the standard value for drinking set by WHO (2008), i.e. 214.3 μM, and DOE (2008), i.e. 28.6 μM. For the middle-stream and downstream zones, which were influenced by the tidal event from sea water, the Marine Water Quality Criteria for the ASEAN Region (AMWQC) was used as a guideline for the livelihood of aquatic living organisms. In several occasions, the levels of nitrite at the middle-stream and downstream exceeded the standard value (i.e. 3.93 μM) set by AMWQC of AWGCME (2004).

High nitrate (18.9 μM) was observed at upstream, and it eventually decreased slightly along the middle-stream (9.9 μM) and downstream (3.5 μM) of the river. Leachates from fertiliser consumption in the orchard plantations eventually ended up in the river via groundwater runoff. As Pinang River, Balik Pulau is the source of freshwater supply for drinking, the nitrate level in it should not exceed 3571.4 μM (WHO 2008), which is a recommended value for drinking water to protect against methaemoglobinaemia that can cause blue baby syndrome. In Malaysia, the recommended value of nitrate level for drinking water should be less than 500 μM (DOE 2008). A high nitrate level was recorded at the upstream and occasionally at the middle-stream as well. Besides agricultural practices, the lack of sewage treatment and wastewater treatment from residential areas and aquaculture ponds were the main contributors to the high levels of nitrate at these stations. Low nitrate levels were recorded at Station 7 that were probably caused due to the dilution by seawater. Sometimes, the concentrations of nitrate at Station 3 to Station 7 exceeded the accepted levels (i.e. 4.29 μM) set by AMWQC.

Among all the sampling stations, the middle-stream indicated to have high ammonia levels (mean of 2.2 μM). Even though the water catchment area at the upstream had received runoff from the agricultural land, surprisingly very low concentrations of ammonia were recorded at upstream. However, the ammonia levels at the middle-stream and downstream exceeded 5 μM set by AMWQC. Probably, direct discharges from domestic wastes (Bellos *et al*. 2004), aquaculture effluent (Khairun 2004) and the leaching of fertiliser usage from oil palm plantations (Hishamudin *et al*. 1987) contributed to the high ammonia levels. The low ammonia level at Station 7 in the sea was due to the effect of water dilution during the high tide.

The ortho-phosphate level was found to be low at the upstream with the mean value of 0.7 μM. Agricultural practices at the upstream did not affect the overall river water quality. However, the middle-stream (4.2 μM) and the downstream (2.1 μM) showed high ortho-phosphate levels that exceeded the level (1.41 μM) set by AMWQC. The results from this study indicate that Pinang River is being polluted by human sewage from domestic areas. In addition, the usage of detergents in household activities (Ntengwe 2006), the high phosphorus content in fertilisers for oil palm plantations (Hishamudin *et al*. 1987) and prawn faeces in aquaculture effluent (Thakur & Lin 2003) are also other factors that contributed to the increase of ortho-phosphate concentrations.

## CONCLUSION

The application of non-supervised ANN by using the physical and chemical parameter data identified the pollution zones of Pinang River, Balik Pulau. The high level of nitrate originated from the water catchment area due to the usage of fertilisers in the orchard plantations. In general, the distribution of BOD_5_, TSS, nitrite, ammonia and ortho-phosphate was very distinct at middle-stream, where wastewater from houses, oil palm plantations and aquaculture ponds were directly discharged into the river. Furthermore, high levels of ammonia, BOD_5_ and TSS were recorded at the downstream end of the river. The impact of anthropogenic activities in Pinang River, Balik Pulau could lead to the deterioration of water and could pose a serious problem for future generations.

## Figures and Tables

**Table 1:** Variation of land-use activities along the Pinang River, Balik Pulau at the sampling stations with the Global Positioning System (GPS) coordinates

Station	Location	Land use activities	GPS	River zone
S1	Agricultural area (Hilly water catchment area)	Primary forest and orchard plantation	5° 23′33.90″N 100° 13′4.17″E	Upstream
S2	Agricultural area (Foothill water catchment area)	Small villages and orchard plantation	5° 23′59.36″N 100° 12′50.48″E	Upstream
S3	Domestic area	Houses and small cottage industry	5° 23′35.38″N 100° 12′0.28″E	Middle-stream
S4	Agricultural area	Oil palm plantation	5° 23′24.78″N 100° 46′0.57″E	Middle-stream
S5	Aquaculture area	Aquaculture ponds	5° 23′31.48″N 100° 11′42.85″E	Middle-stream
S6	Estuary	Mangrove vegetation	5° 23′30.03″N 100° 11′13.71″E	Downstream
S7	Open Sea	Open to Strait of Malacca	5° 23′29.51″N 100° 10′41.65″E	Downstream

**Table 2:** Physico-chemical variables with minimum, maximum and mean values of Pinang River, Balik Pulau from October 2007 until October 2008 at low and high water levels during both spring and neap tides.

Parameter	Upstream			Middle-stream			Downstream		
		
	Min	Max	Mean	Min	Max	Mean	Min	Max	Mean
Temperature (°C)	22.0	26.3	24.4	21.9	33.0	28.5	25.4	33.5	29.6
pH	4.7	9.0	7.0	4.9	9.8	7.4	6.3	9.8	8.0
DO (mg/L)	6.3	9.1	7.9	1.0	10.5	6.7	1.7	10.6	7.0
BOD (mg/L)	0	6.8	1.4	0	12.6	4.1	0	13.9	3.7
Salinity (ppt)	0	0	0	0	31.0	9.8	2.0	34.0	24.5
EC (μMHOS)	10.0	35.0	20.1	20.0	48000.0	17047.2	210.0	50000.0	36021.9
TSS (mg/L)	0	70.0	14.9	0	2366.7	136.9	0	1300.0	244.3
Nitrite (μM)	0	1.2	0.3	0	18.1	1.6	0	19.6	0.9
Nitrate (μM)	7.4	41.4	18.9	0.3	44.7	9.9	0	30.6	3.5
Ammonia (μM)	0	1.8	0.4	0	19.6	2.2	0	11.8	1.0
Ortho-phosphate (μM)	0	2.3	0.7	0	34.2	4.2	0	8.2	2.1
